# Association between biomarkers of redox status and cytokines with different patterns of habitual physical activity in eutrophic and overweight/obese preschoolers: multivariate analysis of a cross-sectional study

**DOI:** 10.1186/s12889-023-17295-y

**Published:** 2023-11-28

**Authors:** Ângela Alves Viegas, Thiago Santos, Juliana Nogueira Pontes Nobre, Jousielle Márcia dos Santos, Vanessa Kelly da Silva Lage, Amanda Cristina Fernandes, Marco Fabrício Dias Peixoto, Rosane Luzia De Souza Morais, Alessandro Sartorio, Vanessa Amaral Mendonça, Ana Cristina Rodrigues Lacerda

**Affiliations:** 1 Multicenter Postgraduate Program in Physiological Sciences (PPGMCF), Federal University of the Jequitinhonha and Mucuri Valleys (UFVJM), Diamantina, Minas Gerais Brazil; 2 Integrated Center for Research and Postgraduate Studies in Health (CIPq Saúde), Federal University of the Jequitinhonha and Mucuri Valleys (UFVJM), Diamantina, Minas Gerais Brazil; 3 Postgraduate Program in Animal Biology (PPGBA), Federal University of the Jequitinhonha and Mucuri Valleys (UFVJM), Diamantina, Minas Gerais Brazil; 4 Postgraduate Program in Rehabilitation and Functional Performance (PPGReab), Federal University of the Jequitinhonha and Mucuri Valleys (UFVJM), Diamantina, Minas Gerais Brazil; 5 Postgraduate Program Health, Society and Environment (PPGSaSA), Federal University of the Jequitinhonha and Mucuri Valleys (UFVJM), Diamantina, Minas Gerais Brazil; 6 Division of Auxology and Metabolic Diseases & Experimental Laboratory for Auxo-endocrinological Research, Istituto Auxologico Italiano, IRCCS, Piancavallo-Verbania, Italy

**Keywords:** Oxidative stress, Accelerometry, Childhood, Body composition, Triglycerides, Leptin, sTNFRs

## Abstract

**Background:**

Although it is well known that obesity is frequently associated with reduced levels of habitual physical activity (HPA), which contributes to determining severe oxidative stress and inflammatory state, this association is however unknown in preschoolers so far. This study aimed to investigate the association between biomarkers of redox status and cytokines with different patterns of HPA according to the adiposity of preschoolers.

**Methods:**

A cross-sectional study was conducted in 50 preschoolers (25 overweight/obese, OW/OB and 25 eutrophic, EU), matched for age, sex, economic level, and maternal education. Total antioxidant capacity (TAC), superoxide dismutase (SOD) and catalase (CAT) activities, substances reactive to thiobarbituric acid (TBARS), soluble tumor necrosis factor receptors (sTNFRs), and leptin levels were evaluated. HPA levels were evaluated by accelerometry (ActiGraph GT9X accelerometer). Correlation, multiple linear regression, and partial least squares regression analysis were used to determine the association between redox status biomarkers and cytokines with different patterns of HPA (HPA level, bouts of moderate to vigorous physical activity [MVPA], and multivariate pattern of HPA) in EU and OW/OB preschoolers.

**Results:**

OW/OB preschoolers had lower CAT activity, higher levels of TAC, TBARS, and cytokines, and similar levels of HPA to EU preschoolers. In EU preschoolers, SOD activity exhibited a stronger negative association with moderate intensity ranges of HPA (R^2^ = 0.18), and negative correlation with sTNFRs (r = -0.40 to -0.46). TBARS had a stronger positive association with ranges of light intensity in the multivariate pattern of HPA (R^2^ = 0.10). In OW/OB preschoolers, the HPA multivariate associative pattern was predominantly from vigorous intensity ranges. Thus, SOD activity had a positive association with the multivariate pattern of HPA (R^2^ = 0.38) and MVPA bouts (β [95% CI] = 0.457 [0.0026. 0.0576]). TAC had a negative association with the multivariate pattern of HPA (R^2^ = 0.38) and MVPA bouts (β [95% CI] = -0.718 [-0.0025. -0.0003]). Additionally, leptin levels were lower in OW/OB preschoolers engaged in vigorous physical activity (VPA) (8000–9999 counts/min) for longer periods of time.

**Conclusion:**

The results of this study indicate that OW/OB preschoolers have higher levels of oxidative stress biomarkers and pro-inflammatory cytokines compared to EU preschoolers. Moreover, VPA may exert antioxidative and anti-inflammatory effects in OW/OB preschoolers.

**Supplementary Information:**

The online version contains supplementary material available at 10.1186/s12889-023-17295-y.

## Background

The redox imbalance is a common consequence of obesity [1] during adulthood and childhood [2–6]. However, the relationship between physical activity (PA) levels, redox status, and inflamation during preschool age is not yet completely elucidated. The development of adipose tissue alters substantially over the preschool years; at approximately two years old, adipose tissue declines and begins to increase substantially at approximately six years of age, which is known as adiposity rebound [7]. The redox system is still immature in preschoolers, and evidence suggests that they may have a redox imbalance characterized by high levels of free radicals and low levels of antioxidant enzymes [8–10]. Another marked difference between adults and children at preschool age is the inflammatory responses. The visceral adipose tissue of obese and non-obese children does not express some inflammatory genes that are commonly expressed in adulthood [11]. Additionally, Ouyang et al., [12] evaluated the inflammatory profile between obese and EU preschoolers and found a significant difference only in serum soluble TNF receptors (sTNFRs) levels between these groups. However, there is a lack of studies analyzing the association between regular PA levels with biomarkers of redox status and inflammatory cytokines in EU and OW/OB preschoolers [3]. Regular PA practice exerts anti-inflammatory effects and an improvement in redox status balance, whereas obesity is linked to an imbalance in redox status and pro-inflammatory status [13]. Studies in older children indicate that these positive effects of PA on redox status balance and inflammation may occur primarily through improvements in the antioxidant defense system [14] and anti-inflammatory cytokines [15].

Robust evidence has shown that the anti-inflammatory effects of regular PA practice in adults involve leptin and Tumor Necrosis Factor (TNF) regulation [16–19]; however, this link remains poorly understood in children. In addition, numerous confounding factors, such as the nature of the PA, prematurity, low birth weight, malnutrition, and disorders hampering growth, may significantly impact the redox status balance and inflammatory biomarkers in children [6, 20–22]. Of note, the most frequent limitation of studies looking into the impact of PA on redox status balance and inflammatory biomarkers in children is the difficulty of precisely assessing the type, intensity, volume, and frequency of PA [23–27]. PA is sporadic in preschool children [28], since preschoolers exhibit a variety of patterns of habitual PA (HPA) throughout the day that vary in intensity, volume, and frequency [24, 25]. Thus, measurements of children’s PA levels (e.g., using accelerometry) should consider the multivariate pattern of HPA including the entire spectrum of intensities [26] and the accumulation of MVPA in multiple daily bouts [27]. To the best of our knowledge, only one study evaluated the redox status balance of children according to PA levels using accelerometers, a gold-standard method for measuring PA levels; however, this study did not involve preschoolers [29].

Thus, this study aimed to investigate the association between biomarkers of redox status and cytokines with different patterns of HPA according to the adiposity of preschoolers. First, we analyzed biomarkers of redox status and cytokines (leptin and sTNFRs) levels according to HPA levels using accelerometers in EU and OW/OB preschoolers. Moreover, we investigated the association between redox status and multivariate HPA pattern according to adiposity, adjusted for age, sex, protein/carbohydrate ratio (ptn/carbo), triglycerides, sTNFRs and leptin. Finally, we examined the association between redox status and multivariate HPA pattern based on adiposity and daily bouts of at least 2 min of MVPA.

## Methods

### Study design and participants

This is a quantitative, exploratory, and cross-sectional study. The study was approved by the Research Ethics Committee of the Universidade Federal dos Vales do Jequitinhonha e Mucuri (UFVJM) (Protocol: 2.773.418), with written informed consent parental and participant assent. Preschoolers aged 3 to 5 years, from public schools in the municipality of Diamantina – Minas Gerais, Brazil, were eligible. Recruitment and data collection were done in the second half of 2019.

The sample size was estimated using GPower® (Franz Faul, Universitat Kiel, Germany), version 3.1.9.2. F-tests were used for the multiple linear regression models. Given the scarcity of studies on the association between redox status and PA in children, the sample size was calculated using data on SOD activity from Chaki et al. [30] with prepubertal and pubertal children (9 to 11 and 15 to 17 years, respectively). Age and body mass index (BMI) were predictors of SOD activity. The model’s R^2^ increased after PA. Thus, the partial R^2^ for PA’s increase in SOD activity was equal to 0.27, while the effect size (f^2^) was equal to 0.36. Thus, the sample size was estimated at 25 volunteers, considering a power of 0.80 and an alpha error of 5%.

Initially, 56 children were recruited for this study. Exclusion criteria were: preterm births, low birth weight, complications in pregnancy and childbirth, signs of malnutrition or diseases that interfere with growth and development, use of medication or vitamin/antioxidant supplementation, infectious process (such as fever, flu, diarrhea) or vaccinations in the last 30 days before recruitment.

Of the initial cohort, fifty children were considered eligible for the study and were divided into two subgroups: 1-overweight/obese (OW/OB) preschoolers (n = 25) with a BMI ≥ 97th percentile (z-score > + 2), using the WHO reference charts [31]. 2-eutrophic (EU) preschoolers (n = 25) with BMI between 15th and 85th percentile (-1 < z-score < + 1) [31] matched for sex, age, economic level, and maternal education. The OW/OB preschooler’s group consisted of eight overweight and seventeen obese children. As children from the OW/OB group were recruited, EU children of the same sex from the same classroom were also recruited so that the groups were composed of children of very similar ages and similar socio-environmental realities (later verified by comparing the economic level and maternal education, between the groups – Table 1). The maximum age difference between the groups was four months.

### Procedures

#### Anthropometric data and body fat mass

The BMI was calculated as the ratio between weight (kg) and height (m) squared, and classification was performed according to the World Health Organization guidelines [31, 32] (WHO, 2006; De Onis et al., 2010). WHO Anthro software version 3.2.2 (Geneva, Switzerland) was used to calculate BMI according to age and sex, expressed in z-scores. Fat mass and fat free mass were assessed by Dual Energy Radiological Absorbometry (DEXA) using pediatric mode (Pediatric medium scan mode software, Lunar Radiation Corporation, Madison, Wisconsin, USA, model DPX). The instrument was properly calibrated and the scans were analyzed by a trained technician. The fat mass index (FMI) and fat free mass index (FFMI) was calculated as the ratio between the fat mass or fat free mass and the height squared [33].

#### Economic level

The Brazilian Economic Classification Criterion was used to verify the economic level of the families. The questionnaire stratifies the general economic classification from A1 (high economic class) to E (very low economic class) and takes into account the family’s assets, the education of the householder, and housing conditions such as running water and paved streets [34].

#### Blood collection

After 8 to 12 h of fasting, 10 mL of venous blood was drawn and distributed in sterile tubes with and without anticoagulants for laboratory analysis of redox status biomarkers, cytokines, and lipid profile. Plasma and red blood cells were aliquoted after the samples were centrifuged immediately after blood collection and stored at -80 °C until the time of analysis.

Blood aliquots from 25 EU and 25 OW/OB preschoolers were thawed and homogenized in PBS (0.05 M, pH 7.4) at 0–4 ºC over 3 min for analyses of redox status biomarkers, cytokines, and lipid profile.

#### Redox status biomarkers

The redox status was determined by measuring the concentration of total antioxidant capacity (TAC), activity of the antioxidant enzymes superoxide dismutase (SOD) and catalase (CAT), and the thiobarbituric acid reactive substances (TBARS), in erythrocyte lysate [35]. Protein concentration of samples was determined by the Bradford method [36] using bovine serum albumin (BSA) (1 mg/mL) as standard. TAC was measured by ferric reducing antioxidant power (FRAP) [37]. The samples were analyzed in duplicate, in a microplate reader (SpectranMax®190, 34 Molecular Devices, USA), at 593 nm. Total antioxidant capacity was determined from the standard curve of known FeSO4 concentrations and normalized by the amount of protein in the sample. Results were expressed in micrograms FeSO4 per milligram of protein. SOD activity (EC 1.15.1.1) was determined by the ability of SOD to inhibit pyrogallol autoxidation, where 1U = 50% inhibition of pyrogallol autoxidation [38]. Reading was carried out for 4 min at 37ºC in a microplate reader (SpectranMax®190, Molecular Devices, USA), at 420 nm. SOD activity was expressed in units (U) per milligram of protein. CAT activity (EC 1.11.1.6) was measured according to the enzyme activity variation (ΔE) for one minute at 25 °C in a spectrophotometer at 240 nm (Libra S22 spectrophotometer, Bochrom, UK) [39]. Results were expressed in ΔE/min per milligram protein. TBARS levels were obtained by reacting thiobarbituric acid with malondialdehyde (MDA) to determine lipid peroxidation in cells and tissues [40]. The concentration of TBARS was determined after reading on the microplate reader (SpectranMax®190, Molecular Devices, USA) at 532 nm and according to the standard curve of known concentrations of MDA (1,1,3,3-tetramethoxypropane) (Sigma, USA). TBARS level was expressed in nanomoles of MDA per milligram of protein.

#### Cytokines: sTNFRs and leptin

Plasma sTNFR1, sTNFR2 and leptin levels were measured using conventional sandwich ELISA kits (DuoSet, R&D Systems, Minneapolis, MN, USA) according to the manufacturer’s instructions. The detection limits were 5 pg/mL for all kits.

#### Lipid profile

The lipid profile (triglycerides, total cholesterol, and its fractions – HDL and LDL) was evaluated in a single certified clinical laboratory. The lipid and lipoprotein levels were classified according to the recommendations on cardiovascular health and risk reduction in children and adolescents [41]. In exploratory analyses, total cholesterol and its fractions did not show significant correlations. Therefore, the results of this classification were presented only for triglyceride values. Thus, for children aged 0 to 9 years, the cutoff points for values ​​considered acceptable, borderline to high, and high for triglycerides are: <75, 75–99, and ≥ 100 mg/dL, respectively. The cut-off points represent the 75th and 95th percentile for determining borderline high and high values.

#### Dietary intake

Dietary intake was evaluated by a three-day food record. All records were filled out by one of the children’s parents. They were previously instructed to register detailed information on food, fluid, and supplement intake [42, 43]. All information was obtained from two non-consecutive habitual days (Monday through Friday) and one non-habitual day (Saturday or Sunday). The daily caloric intake of the preschoolers was obtained from the food records using the DietPro 5i software (A.S. Sistemas, Viçosa, Minas Gerais, Brazil). The DietPro software has several food databases available for nutrient calculations allowing to choose the more suitable database for the data. For the present study, the TACO (Tabela Brasileira de Composiço de Alimentos) [44] database was chosen since it is considered the best Brazilian nutrient database. Additionally, we updated the program database with the nutritional information of some processed foods. The average calorie intake was estimated using the Multiple Source Method (MSM), which takes into account intra- and inter-individual variability (https://msm.dife.de). The Recommended Dietary Allowances (RDA) were utilized as a guide for the macronutrient intake of preschoolers (protein = 1.05 to 0.95 g/kg/day; carbohydrates = 130 g) [45].

#### Habitual physical activity

The different patterns of HPA in preschoolers were measured by accelerometers (ActiGraph GT9X accelerometer - Pensacola, FL). The accelerometers were attached to the children’s hip for up to seven days [46]. Accelerometers were set to record accelerations at 60 Hz and records were analyzed at five-second epochs to capture low and high intensities PA [47] using ActiLife v.6.13.3 software (ActiGraph, Pensacola, FL, USA). In all analyses, consecutive periods ≥ 20 min with zero counts/min (cpm) were considered non-use time [48]. Moreover, records were considered valid if the period of use was ≥ 570 min/day [49]. The average of 3 days’ records was included in the analyses [46].

For descriptive statistics and bivariate correlation analysis, we used time (min/day) at sedentary behavior (SED behavior: ≤819 cpm) and at traditional PA intensities (light-intensity – LPA: 820–3907 cpm; moderate-intensity – MPA: 3908–6011 cpm; vigorous-intensity- VPA: ≥ 6612 cpm) for the magnitude vector as proposed by Butte et al. [50].

In addition, light, moderate and vigorous intensities (LMVPA: ≥ 820 cpm) and moderate to vigorous intensity (MVPA: ≥ 3908 cpm) were used to determine if children met the recommended HPA levels (children under 5 years: ≥180 min LMVPA and ≥ 60 min MVPA daily; children aged 5 and over: ≥ 60 min MVPA daily) [51]. Children who met the minimum HPA levels were classified as sufficiently active, and those who did not were classified as insufficiently active. All children over 5 years of age classified as sufficiently active also achieved 180 min or more of LMVPA. We also looked pattern of accumulated HPA in daily bouts (at least 2 min of MVPA bout with a drop time of 2 min at other intensities). For the analyses, the number (frequency) and average (min) of the total time in bouts per day were used [52].

In addition, a broad intensity spectrum was used to assess the multivariate associative pattern of HPA with redox status biomarkers. A dataset using 23 HPA variables of total time (min/day) was created to capture movement at narrow intensity ranges across the full spectrum of activities: 0–99, 100–249, 250–499, 500–999, 1000–1499, 1500–1999, 2000–2499, 2500–2999, 3000–3499, 3500–3999, 4000–4499, 4500–4999, 5000–5499, 5500–5999, 6000–6499, 6500–6999, 7000–7499, 7500–7999, 8000–8499, 8500–8999, 9000–9499, 9500–9999, and ≥ 10,000 cpm [26, 53].

### Statistical analysis

Descriptive analysis was performed to determine the distribution of data for EU and OW/OB groups, as well as for sufficiently and insufficiently active groups. Data were presented as mean ± standard deviation or median and interquartile ranges. The Shapiro-Wilk test and the QQ-plot were used to verify the normal distribution of the data and the Levene test to verify the homoscedasticity. To compare EU and OW/OB groups, the t-test for quantitative variables with equal assumed and non-assumed variances according to the result of the Levene test was used, and Chi-square for categorical variables. The Mann-Whitney *U* test was used for variables with non-normal distribution to compare sufficiently and insufficiently active children within the EU and OW/OB groups. Pearson’s or Spearman’s bivariate correlations were used according to the normality of the data in each group (EU and OW/OB groups, as well as sufficiently and insufficiently active groups), to verify the associations between HPA (traditional PA intensities), adiposity, redox status markers, cytokines, triglycerides and ptn/carbo ratio. The correlation between the average daily total time of at least 2 min of MVPA bouts and traditional PA intensities was also verified.

Multivariate pattern analysis, performed on R (v. 3.6.2) (Supplementary material - script), was used to verify the associations with the distribution of time spent at each PA intensity in a more detailed spectrum. Partial least squares (PLS) regression analysis [26] was used to determine the multivariate pattern of HPA associated with redox status markers (dependent variables), including all 23 HPA variables as independent variables. Before PLS regression analysis, residuals from linear regression models were obtained using all oxidative stress markers as dependent variables in separate models and all covariates (age in months, sex, ptn/carb ratio, triglycerides, sTNFRs, and leptin) as independent variables. Residuals were used in the PLS to thus adjust the models for confounding variables [54].

PLS regression decomposes the independent variables into orthogonal linear combinations while simultaneously maximizing the covariance with the dependent variable, completely eliminating the collinearity between the variables. PLS adequately handles a small sample size in the face of a large number of independent variables. For the PLS, all variables were centered and standardized for unit variance. After PLS regression analysis, we performed a *leave one out* cross-validation analysis (also known as full cross validation) as it is typically considered the most robust cross validation especially for small samples [55, 56]. This analysis was used to optimize the predictive performance of models and select the component variables.

From the first local minimum, only the first or first two components were selected in the PLS regression models for the redox status biomarkers [57, 58]. The *Selectivity ratio* (SR) was determined by the ratio between the predictive variance and the residual variance for each HPA variable to reveal the strongest associations with the outcome among a high number of variables [59]. The results were presented in an SR plot indicating the positive and negative associations with the redox status biomarkers, in addition to the explained variance (R^2^) of each model. The SR plots display quantitatively the HPA variables according to their predictive and discriminatory importance for redox status biomarkers. Confidence intervals were constructed around each SR and used to assess the significance of the SR for each HPA variable [54]. Thus, the associations observed in the PLS allow for identifying the importance of each variable (band) of the broad spectrum of HPA intensities to predict the outcome while simultaneously considering all HPA intensities in a single model.

The association between at least 2-min bouts of MVPA (mean total time per day) and redox status markers were also analyzed using linear regression adjusted for age in months, sex, ptn/carbo ratio, triglycerides, soluble TNF and leptin. All analyses, except PLS, were performed using SPSS 22.0 (IBM SPSS Statistics for Windows, Armonk, NY; IBM Corp., USA), considering a significance level of 5%.

## Results

Table 1 shows the characteristics of EU and OW/OB groups. Maternal education, economic level, gestational age at birth, and birth weight, were not different between EU and OW/OB groups. As expected, the OW/OB group had higher BMI, fat mass, FMI, fat free mass and FFMI compared to the EU group. Compared with EU group, OW/OB presented higher TAC and TBARS levels and lower CAT activity. Caloric intake, carbohydrate, and protein consumption were higher in OW/OB than EU group. However, when compared with the RDA recommendations (carbohydrate intake = 130 g; protein intake = 1.05–0.95 g/kg/day) [43], both EU and OW/OB groups had a higher daily carbohydrate and protein intake (EU group: 3.1 g/kg/day ± 0.61; OW/OB group: 2.4 g/kg/day ± 0.58). In addition, both EU and OW/OB groups had individuals with triglyceride concentration above the acceptable level, and therefore considered metabolically unhealthy for their age. OW/OB group had higher levels of leptin, sTNFR1, and sTNFR2 compared to EU group. When comparing biomarkers of redox status and cytokines between OW and OB, only leptin levels were higher in OB (OW = 1124.32 [995.75–1252.56]; OB = 1320.96 [1275.31 -1490.30], U = 19.00, p = 0.00).

**Table 1:** Characteristics of EU (n = 25) and OW/OB (n = 25) preschoolers

	EU group	OW/OB group	X^2^/T	p
Sex *n (%)*			0.00*	1.00
Female	14 (56)	14 (56)		
Male	11 (44)	11 (44)		
Age in months *m ± sd*	62.6 ± 8.2	62.3 ± 7.5	0.14	0.88
Maternal education *n (%)*			4.33*	0.22
Elementary	4 (16)	5 (20)		
High school	17 (68)	12 (48)		
Graduated	4 (16)	8 (32)		
Economic level *n (%)*			4.16*	0.38
B	4 (16)	10 (40)		
C	19 (76)	13 (52)		
D, E	2 (8)	2 (8)		
Gestational age *(weeks) m ± sd*	39.2 ± 1.5	39.1 ± 1.2	0.30	0.76
Birth weight *(kg) m ± sd*	3.2 ± 0.4	3.3 ± 0.4	-0.85	0.39
Body composition (*kg/m*^*2*^*or kg*) *m ± sd*				
BMI	15.2 ± 0.6	21.9 ± 2.1	-12.29	**< 0.01**
Fat mass	3.9 ± 1.1	10.7 ± 3.0	-10.40	**< 0.01**
FMI	3.1 ± 0.7	7.8 ± 1.7	-6.17	**< 0.01**
Fat free mass	11.6 ± 1.5	15.0 ± 2.6	-5.50	**< 0.01**
FFMI	9.4 ± 0.7	11.0 ± 1.0	-6.23	**< 0.01**
Redox status *m ± sd*				
TAC (FeSO4.L^− 1^.mg protein^− 1^)	0.50 ± 0.07	0.60 ± 0.065	-4.99	**< 0.01**
SOD (U/mg protein)	9.47 ± 2.47	8.22 ± 2.18	1.90	0.06
CAT (ΔE/min.mg protein^− 1^)	2.15 ± 0.93	1.59 ± 0.73	2.35	**0.02**
TBARS (nmol MDA/mg protein)	1.96 ± 0.58	2.46 ± 0.61	-2.92	**< 0.01**
Dietary intake *m ± sd or %*				
Calories (Kcal)	1491.4 ± 221.6	1643.6 ± 195.6	-2.54	**0.01**
Carbohydrate *gr*	205.2 ± 29.2	224.7 ± 35.8	-2.09	**0.04**
%Carbohydrate	55.1 ± 3.4	54.5 ± 4.6	0.50	0.61
Protein *gr*	58.7 ± 13.3	71.1 ± 15.8	-2.95	**< 0.01**
%Protein	15.7 ± 2.4	17.2 ± 3.0	-2.04	**0.04**
Fat *gr*	49.7 ± 9.0	52.8 ± 7.9	-1.26	0.21
%Fat	29.9 ± 3.0	29.0 ± 3.9	0.91	0.36
Ptn/Carbo	0.28 ± 0.05	0.32 ± 0.07	-1.84	0.07
Lipid profile (*mg/dL*) *m ± sd*				
Total cholesterol	159.2 ± 23.8	168.6 ± 24.7	-1.37	0.17
HDL	50.3 ± 7.2	52.0 ± 6.7	-0.87	0.38
LDL	94.1 ± 20.2	102.9 ± 18.5	-1.61	0.11
Triglycerides	73.6 ± 27.4	68.0 ± 38.4	0.60	0.55
Acceptable *n (%)*	14 (56)	15 (60)	0.61*	0.73
Borderline to high *n (%)*	6 (24)	7 (28)		
High *n (%)*	5 (20)	3 (12)		
Cytokine *(pg/mL) m ± sd*				
sTNFR1	132.9 ± 88.6	224.1 ± 148.6	-2.63	**0.01**
sTNFR2	1746.2 ± 389.2	2021.7 ± 336.5	-2.67	**0.01**
Leptin	565.7 ± 412.2	1296.3 ± 278.5	-7.34	**< 0.01**
HPA *(min) m ± sd*				
SED	393.0 ± 45.7	398.8 ± 42.7	-0.46	0.64
LPA	192.4 ± 37.8	186.3 ± 32.8	0.61	0.54
MPA	38.4 ± 11.1	42.3 ± 9.4	-1.31	0.19
VPA	20.8 ± 7.2	19.3 ± 5.8	0.82	0.41
MVPA	59.3 ± 15.2	61.6 ± 14.2	-0.55	0.58
Nº bouts ≥ 2 min	50.1 ± 13.5	52.9 ± 11.9	-0.78	0.43
Total bouts time ≥ 2 min	134.2 ± 38.0	141.7 ± 33.6	0.87	0.46
Valid total wear time	648.3 ± 26.9	643.4 ± 33.3	0.57	0.57
HPA classification *n (%)*			0.32*	0.56
Insufficiently active	12 (48)	10 (40)		
Sufficiently active	13 (52)	15 (60)		

EU: Eutrophic. OW/OB: Overweight/Obese. Elementary: up to the ninth school year. High School: three years of intermediate/high school. Graduated: university education. Economic level: high income - A and B, average income - C; low income - D and E. BMI: Body Mass Index. FMI: Fat Mass Index. FFMI: Fat Free Mass Index. TAC: Total antioxidant capacity. SOD: Superoxide dismutase. CAT: Catalase. TBARS: Substances seactive to thiobarbituric acid. Ptn/carbo: protein/carbohydrate ratio. HDL: High Density Lipoprotein. LDL: Low Density Lipoprotein, sTNFR1: Soluble Tumor Necrosis Factor Receptors (1) sTNFR2: Soluble Tumor Necrosis Factor Receptors (2) HPA: regular physical activity. SED: sedentary behavior. LPA: light physical activity. MPA: moderate physical activity. VPA: vigorous physical activity. MVPA: moderate to vigorous physical activity. *Nominal or ordinal variables whose association with weight status was analyzed by X^2^. Quantitative variables with normal distribution were analyzed by the T test. m ± (sd): mean ± (standard deviation). Values ​​in bold show p value < 0.05.

Supplementary Table 1 shows the HPA levels of EU and OW/OB groups. HPA levels were similar between EU and OW/OB groups in all traditional ranges, including time of SED behavior, LPA, MPA, VPA, and MVPA. Moreover, accumulated HPA, i.e., number and time of MVPA bouts ≥ 2 min, were also similar between groups. However, the pattern of accumulated HPA was different between EU and OW/OB groups (Table 2). Only OW/OB group presented a strong positive correlation between total time of bouts at MVPA and VPA intensities. Thus, especially for OW/OB preschoolers, the longer the duration of HPA bouts at MVPA intensity, the longer the duration of HPA bouts at VPA intensity, and vice versa. Only EU preschoolers showed a strong negative correlation between total time of MVPA bouts and SED behavior and a strong positive correlation between total time of MVPA bouts and total time of HPA at LPA intensity. Interestingly, only in OW/OB group a positive correlation between FMI and SED behavior was detected.

The correlation between traditional intensities of PA, total time of bouts at MVPA, redox status biomarkers and cytokines are reported in Table 2. In EU preschoolers, SOD activity correlated negatively with traditional intensities of PA (MPA and MVPA), total time of bouts at MVPA and sTNFRs. In OW/OB preschoolers, TAC levels correlated positively with SED behavior and negatively with traditional intensities of PA (LPA, VPA, and MVPA) as well as with the total time of bouts at MVPA. SOD activity correlated positively with traditional intensities of PA (VPA and MVPA) and with total time of bouts at MVPA, and negatively with SED behavior. Therefore, the correlation of SOD activity with MVPA was opposite to that observed in the EU group.

**Table 2:** Correlation between HPA, adiposity, redox status biomarkers and cytokines in EU and OW/OB groups

	1	2	3	4	5	6	7*	8	9*	10	11	12*	13*	14	15*
1. SED		**-0.63**	**-0.54**	**-0.37**	**-0.51**	**-0.55**	0.18	**0.53**	**0.49**	**-0.47**	-0.22	0.02	-0.30	-0.12	0.30
2. LPA	**-0.83**		**0.59**	**0.31**	**0.52**	**0.65**	-0.09	-0.15	**-0.39**	0.20	0.06	-0.11	0.03	-0.01	-0.36
3. MPA	**-0.80**	**0.80**		**0.74**	**0.96**	**0.93**	0.29	0.11	-0.54	0.35	0.19	-0.09	0.15	0.13	-0.18
4. VPA	-0.23	0.17	0.33		**0.89**	**0.83**	0.00	-0.15	**-0.55**	**0.49**	0.25	-0.29	0.00	-0.10	-0.29
5. MVPA	**-0.70**	**0.66**	**0.89**	**0.72**		**0.95**	0.19	-0.05	**-0.57**	**0.43**	0.23	-0.21	0.10	0.04	-0.27
6. Bouts	**-0.76**	**0.77**	**0.92**	**0.60**	**0.96**		0.06	-0.12	**-0.59**	**0.43**	0.19	-0.18	0.05	0.05	-0.34
7. BMI	-0.27	0.32	0.35	0.06	0.28	0.29		**0.79**	-0.03	0.06	-0.06	0.35	0.11	0.04	**0.54**
8. FMI	0.02	0.14	0.02	-0.04	0.00	0.00	**0.54**		0.23	-0.09	-0.04	0.13	0.06	0.01	**0.39**
9. TAC*	-0.10	0.25	0.18	0.04	0.17	0.23	0.31	0.20		**-0.50**	0.00	0.22	-0.11	0.00	0.21
10. SOD	0.34	-0.35	**-0.41**	-0.28	**-0.44**	**-0.39**	0.05	0.22	-0.28		**0.62**	0.03	0.12	-0.22	-0.01
11. CAT	0.31	-0.30	-0.31	-0.11	-0.28	-0.20	0.01	0.24	-0.14	**0.81**		0.18	0.15	-0.07	0.02
12. TBARS	-0.25	0.27	0.21	-0.05	0.13	0.16	0.29	0.12	0.11	-0.04	-0.01		0.15	0.13	0.24
13. sTNFR1*	0.12	-0.04	0.23	0.22	0.25	0.20	-0.21	0.09	0.13	**-0.46**	-0.11	0.01		**0.68**	-0.16
14. sTNFR2*	-0.08	0.08	0.14	0.15	0.17	0.12	-0.21	-0.13	0.26	**-0.40**	**-**0.36	0.05	**0.54**		-0.15
15. Leptin*	0.07	0.13	0.10	0.13	0.09	0.10	**0.51**	**0.64**	-0.01	0.21	0.09	0.32	-0.21	-0.31	

Bivariate correlations in OW/OB (top triangle) and EU (bottom triangle) preschoolers. *Variables with non-normal distribution for which Spearman’s correlation was performed. HPA: habitual physical activity. SED: sedentary behavior. LPA: light physical activity. MPA: moderate physical activity. VPA: vigorous physical activity. MVPA: moderate to vigorous physical activity. BMI: Body Mass Index. FMI: Fat Mass Index. TAC: Total antioxidant capacity. SOD: Superoxide dismutase. CAT: Catalase. TBARS: Substances reactive to thiobarbituric acid. sTNFR1: Soluble Tumor Necrosis Factor Receptors (1) sTNFR2: Soluble Tumor Necrosis Factor Receptors (2) Values in bold show correlation coefficients with p value < 0.05.

**Fig. 1:**
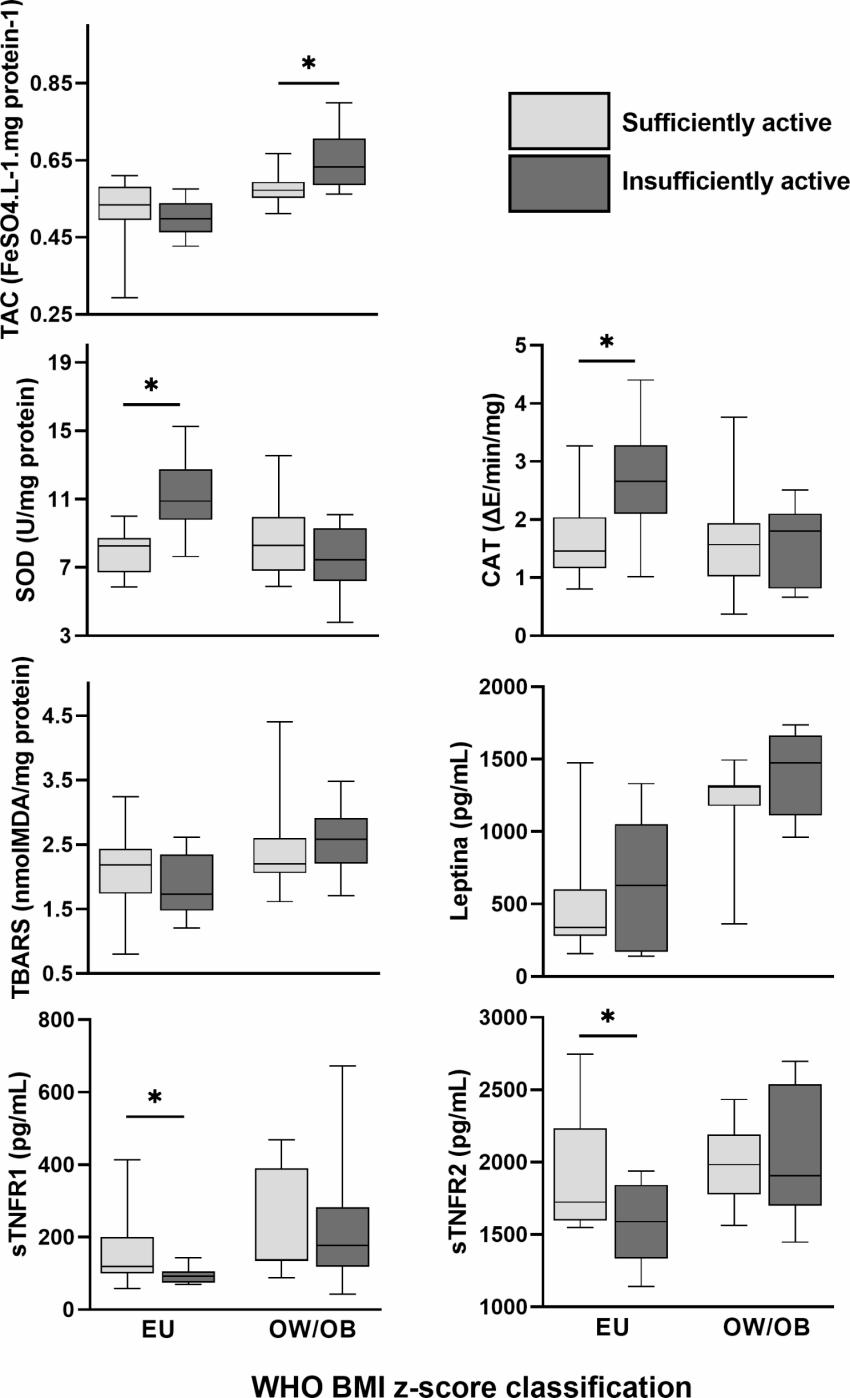
Redox status biomarkers and cytokines levels in EU and OW/OB preschoolers according to HPA levels. TAC: Total antioxidant capacity; SOD: Superoxide dismutase; CAT: Catalase; TBARS: Substances reactive to thiobarbituric acid; sTNFR1: Soluble Tumor Necrosis Factor Receptors 1; sTNFR2: Soluble Tumor Necrosis Factor Receptors 2; WHO: World Health Organization; BMI: Body Mass Index. *p < 0.05

Figure 1 presents the redox status biomarkers and cytokines levels in EU and OW/OB preschoolers according to HPA levels. In the EU group, preschoolers who were insufficiently physically active presented higher SOD and CAT activity and lower levels of sTNFR1 and sTNFR2 than those sufficiently physically active. No differences were found in triglyceride levels and ptn/carbo ratio between EU sufficiently physically active (triglycerides = 66.00 mg/dL [50.00–78.00], ptn/carbo = 0.26 [0.23–0.33]) and insufficiently physically active children (triglycerides = 76.00 mg/dL [48.25-113.75], U = 60.00, p = 0.32; ptn/carbo = 0.29 [0.22–0.38], U = 65.00, p = 0.48). However, a positive correlation between triglycerides and SOD (r = 0.50, p = 0.01) and CAT (r = 0.41, p = 0.04) activities was observed in EU group.

In OW/OB group, preschoolers who were insufficiently physically active presented higher levels of TAC than those sufficiently physically active (Fig. 1). The Ptn/carbo ratio was higher in the insufficiently physically active OW/OB group (0.36 [0.32–0.43]) than in the sufficiently physically active OW/OB group (0.27 [0.24–0.30]; U = 22.00, p = 0.00). Moreover, Ptn/carbo ratio and triglyceride in the OW/OB group presented a positive correlation with TBARS levels (r = 0.45, p = 0.02; r = 0.52, p = 0.01, respectively). Triglycerides also correlated positively with CAT activity (r = 0.44, p = 0.02).

Interestingly, the positive correlation between adiposity and ptn/carbo, and TAC and TBARS were only observed in EU and OW/OB insufficiently physically active preschoolers (Supplementary Table 2). Likewise, the positive correlation of cytokines (sTNFRs and/or leptin) with TAC and TBARS, and negative with SOD activity, was also observed only in EU and OW/OB insufficiently physically active preschoolers (Supplementary Table 2).

**Fig. 2:**
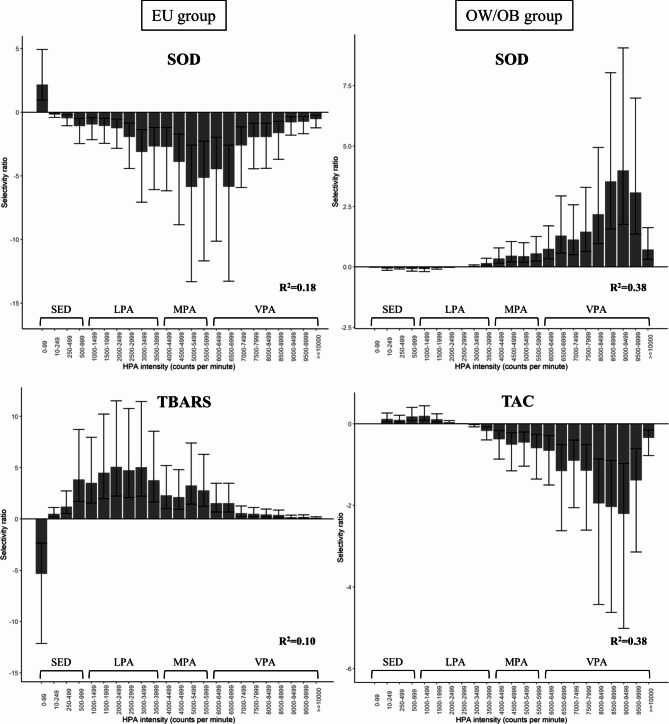
Multivariate pattern of HPA associated with redox status biomarkers in EU and OW/OB preschoolers. The associations are displayed as a Selectivity Ratio (SR) plot. The PLS regression models include 1 component, and age in months, sex, ptn/carbo ratio, triglycerides, sTNFRs, and leptin were adjusted. The SR for each variable is calculated as the ratio of explained to residual variance on the predictive. SOD: Superoxide dismutase; TBARS: Substances reactive to thiobarbituric acid; TAC: Total antioxidant capacity

Figure 2 presents the association between the multivariate pattern of HPA and redox status biomarkers in EU and OW/OB groups. The pattern of HPA of the latent component obtained in each adjusted model explained only the proportion of SOD activity variance in EU (R^2^ = 0.18) and OW/OB (R^2^ = 0.38) groups, the proportion of TBARS variance in EU group (R^2^ = 0.10), as well as the proportion of TAC variance in OW/OB group (R^2^ = 0.38).

In the EU group, the association of the multivariate pattern of the HPA with the SOD activity showed that only the lower intensity band (SED behavior) presented positive SR, while all other bands presented negative SR. The range between 4500 and 6999 cpm showed the most significant importance in this association. Thus, based on the preschooler PA traditional intensities cut-off points, this range of HPA corresponds to the entire MPA spectrum and includes the lower limits of the VPA. However, TBARS had a stronger and negative association with the lowest intensity band of SED behavior (0–99 cpm) and a positive one with light intensity ranges (500 to 3999 cpm) in the multivariate pattern of HPA.

The associations between HPA intensity ranges and SOD activity in the OW/OB group were opposite of those in the EU group. Furthermore, only in the OW/OB group time spent at intensities below 3000 cpm (SED behavior and LPA) was not associated with SOD activity. The range between 8000 and 9999 cpm showed the most significant importance in this association. Thus, based on the preschooler PA traditional intensities cut-off points, this range of HPA corresponds to VPA.

In the OW/OB group, TAC levels were negatively associated with the multivariate pattern of the HPA; however, similar to the results of SOD activity, time spent at intensities below 3000 cpm was not associated with TAC levels. The range between 8000 and 9999 cpm also showed significantly greater importance in this association (Fig. 2). Therefore, based on the multivariate pattern of HPA, the range of VPA in association with SOD activity and TAC in OW/OB preschoolers was determined. Thus, by comparing cytokines and metabolic stressors among OW/OB preschoolers below and above the median time in the range of 8000 to 9999 cpm, those subjects above the median had lower leptin levels (leptin _top 50%_ = 1243.78 [1124.32-1314.12]; leptin _bottom 50%_ = 1438.21 [1220.17-1614.50], difference of medians [95% CI] = 194.43 [9.57–387.20], U = 38.00, p = 0.03). However, considering the entire traditional spectrum of VPA, no differences were found between OW/OB preschoolers below and above the median (leptin _top 50%_ = 1243.78 [1147.86-1311.84]; leptin _bottom 50%_ = 1438.21 [1180.40-1614.50], difference of medians [95% CI] = 194.43 [-4.56-380.20], U = 43.00, p = 0.05). Although the groups according to median time in the range of 8000 to 9999 cpm and the entire traditional spectrum of VPA showed the same medians for leptin, the confidence interval provided a range of more likely values for the difference between the medians in the population [60]. Thus, there was a significant difference in leptin levels between the groups only when we considered the time in the range of 8000 to 9999 cpm.

Finally, in addition to the total volume of HPA analyzed in a multivariate pattern, we verified whether the accumulation of daily bouts of at least 2 min of MVPA was associated with redox status biomarkers in models adjusted for age, sex, ptn/carb, triglycerides, sTNFRs and leptin. Table 3 demonstrates that associations with total time of bouts at MVPA were only observed in OW/OB children and were positive for SOD activity and negative for TAC.

**Table 3:** Association between total time of MVPA bouts and redox status in EU and OW/OB preschoolers

	EU group β (95% CI)	OW/OB group β (95% CI)
Total bouts time ≥ 2 min		
TAC	-0.126 (-0.0012. 0.0007)	**-0.718 (-0.0025. -0.0003)**
SOD	-0.279 (-0.0416. 0.0052)	**0.457 (0.0026. 0.0576)**
CAT	0.113 (-0.0130. 0.0074)	-0.028 (-0.0095. 0.0082)
TBARS	0.214 (-0.0042. 0.0108)	-0.011 (-0.0108. 0.0104)

Multiple linear regression analysis adjusted for age, sex, protein/carbohydrate ratio (ptn/carb), triglycerides, soluble TNF receptors and leptin. β: standardized regression coefficient. CI: confidence interval. TAC: Total antioxidant capacity. SOD: Superoxide dismutase. CAT: Catalase. TBARS: Substances reactive to thiobarbituric acid. Values in bold show p value < 0.05.

## Discussion

To the best of our knowledge, the current study is the frst to report the redox status biomarkers and cytokine levels in preschoolers according to adiposity and HPA levels analyzing a multivariate association pattern between multicollinear HPA and redox status biomarkers. The major findings of this study are: **(i)** OW/OB preschoolers had a worse redox status balance and higher pro-inflammatory cytokines levels compared to EU preschoolers. **(ii)** the only group that showed a negative correlation between SOD activity and sTNFRs was the EU group. **(iii)** sufficiently physically active EU preschoolers had lower SOD activity and higher sTNFRs levels than those insufficiently physically active. **(iv)** the ranges of LMPA in the multivariate HPA pattern was negatively associated with SOD activity and positively with TBARS levels in the EU preschoolers, while it was positively associated with SOD activity and negatively associated with TAC levels in the OW/OB preschoolers. **(v)** in OW/OB preschoolers, the ranges of VPA in the multivariate HPA pattern and MVPA bouts had positive association with SOD activity and negative association with TAC. **(vi)** OW/OB preschoolers who spent more time in the ranges of VPA had lower leptin levels.

### Redox status balance and cytokines levels of preschoolers according to adiposity

OW/OB preschoolers presented lower CAT activity and higher levels of TAC and TBARS than EU preschoolers. The majority of research examined oxidative stress biomarkers in obese children of a wide age range [4–6]. Studies that analyzed SOD and CAT activity in children reported controversial results. Recent investigations have revealed that healthy obese children have a higher SOD activity and a lower or similar CAT activity compared to healthy EU children [5, 61]. However, unhealthy obese children presented similar [5] or lower SOD [4] and CAT activities [5] than EUs. In the present study, triglyceride levels above reference values in the EU and OW/OB groups may have contributed to the differences in CAT activity and similar SOD activity between EU and OW/OB preschoolers [62–64].

As far as TAC is concerned, studies involving preschoolers reported also inconsistent findings, the results varying significantly depending on the age range examined. Studies with children aged 2–11 years found that obese had lower TAC levels than EUs [65, 66]. Studies involving a wider age range, including adolescents, showed higher TAC levels in obese compared to EUs [6, 9, 67]. However, studies that analyzed TAC levels in prepubertal and pubertal individuals separately, revealed that TAC levels in obese were lower than in EU prepubertal [6, 9]. Taken together, these conflicting results indicate possible differences in redox status biomarkers depending on the children’s age, body composition, and health condition.

Other factors, such as the technique used to measure redox status biomarkers, may have influenced the results. The present study evaluated TAC using FRAP, which considers uric acid measurement (reaching 60% of FRAP in human plasma) [37]. A study that evaluated children and adolescents (4.1–17.9 years old) found higher uric acid levels in the obese than in the EU population [68]. However, preschool children seem to produce more uric acid than school children [69]. Since uric acid is an antioxidant, children in the early childhood developmental stage may have more uric acid which protects them from infections [70] and possibly from oxidative stress caused by obesity. In addition, the high energy intake during childhood [71] can also increase uric acid and TAC levels in preschoolers [22, 72]. On the other hand, previous studies that investigated TAC levels in children did not consider their food intake or PA level [6, 9, 65–67].

Similar to the findings from the present study, studies with children and adolescents found that obese have higher levels of TBARS than EUs [4]. Furthermore, we also found a positive correlation between TBARS and ptn/carbo ratio and triglycerides levels only in OW/OB preschoolers. Therefore, in addition to adiposity, obese children with high protein consumption and high levels of triglycerides may have greater lipid peroxidation due to the greater production of reactive oxygen and nitrogen species [22, 73, 74]. In addition, one study found that obese preschoolers and schoolchildren with serum uric acid levels in the highest quartile, considered as a marker of adipogenesis [75], had odds ratios for triglyceride more than twice the odds ratio found in adolescents with the same quartile levels [68]. Therefore, high protein consumption may also be linked to a worse metabolic profile and high oxidative damage in OW/OB preschoolers. In addition to a higher level of oxidative stress biomarkers, OW/OB preschoolers also had higher leptin and sTNFRs levels than EU. Excess weight is related with an increase in leptin levels and a decrease in leptin sensitivity, and these effects seem to occur in preschoolers as well [76].

TNF receptors (TNFR1 and TNFR2) are expressed in human adipose tissue, and their expression and release of soluble ectodomains (sTNFR1 and sTNFR2) increase during obesity [77–79]. However, some studies did not observe differences in TNFR1 levels or circulating sTNFRs between obese and EU children and adolescents [80, 81]. On the other hand, these studies contain significant confounding factors since they evaluated children of varying ages without measuring their PA levels. The only study examining preschoolers indicated that obese 3-year-old girls had elevated amounts of sTNFRs [12], however the results were not adjusted for PA levels.

### Association between redox status biomarkers, cytokines, and different patterns of HPA in EU preschoolers

According to the HPA level, the redox status and cytokines were evaluated in each group. Compared to the insufficiently physically active EU preschoolers, the sufficiently physically active EU preschoolers had lower SOD and CAT activity and higher sTNFRs levels; however, leptin, TAC, TBARS, and triglyceride levels, as well as the ptn/carbo ratio, were not different between the two groups. These and previous findings suggest that sufficiently physically active EU preschoolers have a larger production of reactive oxygen and nitrogen species, a higher consumption of antioxidant enzymes, a decrease in antioxidant enzyme activity, and an increase in sTNFRs release [18, 82, 83].

The results of the two studies that examined SOD activity in EU children aged 6 to 12 according to their PA level were discordant. The first study observed no changes in SOD activity and lipid peroxidation product levels (including MDA) between sedentary and physically active (exercising at least three times per week for at least one year) individuals [84]. The second study found that children performing 4 weeks of daily swimming exercise presented higher SOD activity and lower TBARS levels compared to non-exercised children [85]. Although the type and frequency of PA may alter SOD activity [86], preschoolers were not included in the sample, the metabolic state was not controlled, metabolic-inflammatory parameters were not evaluated, and accelerometry was not used to measure PA levels.

The multivariate pattern of HPA was associated with SOD activity and TBARS in EU preschoolers, and it explained 18% of the negative variation in SOD activity and 10% of the positive variation in TBARS. However, the pattern of HPA accumulated in bouts at MVPA was not associated with SOD activity and TBARS in EU group. This suggests that the multivariate pattern of HPA, rather than the accumulation of MVPA, maybe more important for the negative and positive modulation of SOD activity and TBARS in EU preschoolers, respectively. In addition, the association between HPA levels and SOD activity in the EU group occurred at the lower end of the intensity spectrum, including SED behavior and LPA. Although the importance of each band rose with increasing intensity up to 5499 to 6999 cpm, which is considered MVPA, the association with HPA covered more than one of the traditional intensity domains. Regarding the TBARS, the LPA intensity ranges were more important in the negative association with the multivariate pattern of the HPA. Thus, HPA at the lower end of the intensity spectrum may have contributed to the modulation of SOD activity and TBARS in EU preschoolers by increasing the production of reactive oxygen and nitrogen species, but probably not in a manner required to promote adaptations that augment enzymatic antioxidant reserves [87]. This may have occurred as a result of static light intensity HPA and/or prolonged sitting time, both of which can have adverse health impacts [87, 88]. Therefore, the recommendation for preschoolers to engage in at least 180 min of PA at any intensity (including at least 60 min of MVPA) disregards the potential risks of LPA, depending on how it was performed and the interdependence between intensities to obtain health benefits for children [89]. Thus, achieving minimum HPA levels may not be sufficient for modulating the redox status of preschoolers, but how preschoolers attain the minimum daily HPA target is crucial for this modulation.

In the EU group, children sufficiently physically active presented higher levels of sTNFRs than insufficiently physically active. PA increases reactive oxygen and nitrogen species production, which in turn induces the synthesis of sTNFRs [16–18, 83]. However, we did not observe a linear association between sTNFRs and HPA in the EU group. Therefore, it is possible that the interaction between sTNFRs and HPA in preschoolers depends on a minimum level of HPA. One study found that children aged 5–6 with high levels of PA (e.g., playing, jumping, and running for at least 9 h/day) had lower levels of interleukin [IL]-6, IL-13, TNF and interferon-γ production than children with medium levels of PA (average of 5 h/day). This study found, however, that there were no differences in cytokine levels between highly- physically active children and low- physically active children (less than 2 h/day of PA) [90]. Despite the fact that the researchers did not assess sTNFRs, it is possible that moderately physically active children with high levels of spontaneous inflammatory immune response would also have had higher levels of sTNFRs [91] compared to children with high and low levels of PA, asobserved in the present study. Consequently, the highest concentration of sTNFRs found in sufficiently physically active EU preschoolers appears to be consistent with a median HPA level. According to previous researches [92–94], highly physically active preschoolers spend approximately twice as much time in MVPA on the vertical axis of accelerometry as sufficiently active preschoolers.

In addition, we found a negative correlation between SOD activity and sTNFRs in EU preschoolers. This link has only been studied in adults to date. One study indicated that when MPA was conducted in a 30-minute bout (session), there was a rise in CAT activity 1 h after exercise, accompanied by a return to baseline levels of sTNFR1 [83]. Another study demonstrated a negative correlation between SOD and CAT activity with sTNFR1 levels in middle-aged recreationally active individuals who were not training for or engaging in competitive sports [18]. These studies did not assess sTNFR2 levels, although PA levels may also increase the release of this soluble receptor [19]. A study found that the replacement of 10 min per day of SED behavior with ≥ 1-minute MPA bouts was positively associated with the inflammatory score (interleukin [IL]-2, IL-6, IL-8, IL-10, and adiponectin) in children aged 8 to 9 years. In contrast, when the replacement was by VPA, there was a beneficial association with the inflammatory score [15]. Thus, the inverse correlation between sTNFRs levels and SOD and CAT activity was dependent on the level and/or multivariate pattern of HPA. Therefore, the results of this study suggest that sufficiently physically active EU preschoolers, whose MPA levels were negatively associated with SOD activity, may have greater sTNFRs levels.

### Association between redox status biomarkers, cytokines, and different patterns of HPA in OW/OB preschoolers

There were no variations in antioxidant enzyme activity, cytokines, triglycerides, or TBARS levels between sufficiently and insufficiently physically active in the OW/OB group. The multivariate pattern of HPA, however, explained 24.4% of the positive variation in SOD activity. Moreover, the most significant intensity range (8000 to 9999 cpm) of this association is within the VPA spectrum only in OW/OB preschoolers, and a substantial amount of time in this range has accumulated in bouts. The difference in accumulated time in VPA between EU and OW/OB children may arise even in the absence of PA level variations [25], as found in the current investigation. We also detected a positive correlation between the HPA pattern accumulated in bouts of at least 2 min of MVPA and SOD activity, but only in OW/OB children. Thus in children with obesity, as distinct from those EUs, the HPA pattern may be associated with higher SOD activity. Nasca et al. [24] concluded that obese children appear to engage in shorter exercises followed by increasing frequency at lower intensities of exercise or by rest. Thus, the authors hypothesized that obese children may produce less reactive oxygen species as a result of mitochondrial respiration, resulting in reduced post-exercise oxidative stress. In addition, only EU individuals presented an association between PA and elevated nitric oxide levels [95, 96]. Despite the association, no variations in SOD activity between sufficiently and insufficiently physically active OW/OB preschoolers were identified.

A study with obese children with a mean age of 11 years found that the increase in serum SOD protein was only detected after 24 weeks of PA together with a marked decrease in leptin [97]. Leptin has been demonstrated to affect the generation of oxidative stress indicators, including reduced nitric oxide, increased superoxide, and peroxynitrite, in human endothelial cells and in the endothelium of obese mice [98, 99]. When comparing the levels of metabolic-inflammatory variables between children with excess weight who spent more (top 50%) or less time (bottom 50%) in the range of 8000 to 9999 cpm, those who spent more time in the range of 8000 to 9999 cpm had lower leptin levels. But, no variations in SOD activity were identified.

Similar results were reported in a study of children aged 6 to 8 years, with VPA being inversely associated with leptin in children with higher body fat % but not in those with lower body fat percentages [100]. When we assessed the entire traditional spectrum of VPA and compared children below and above the median, we once again found no difference. Therefore, the analysis of the HPA multivariate pattern allowed us to determine the most significant intensity range not only in the link between HPA and SOD activity but also for leptin in preschool-aged children with excess weight. Thus, it is possible to conclude that OW/OB preschoolers who spent more time in the most significant intensity range (8000 to 9999 cpm) in the association between the multivariate pattern of HPA and SOD activity did not reduce leptin levels sufficiently to demonstrate higher SOD activity than OW/OB preschoolers who spent less time in this range.

Another important result in the present study is that in the OW/OB group, those who were sufficiently physically active had lower TAC levels and lower ptn/carbo ratio than the insufficiently physically active. Thus, the shift in TAC levels according to HPA levels may have been attributable, at least in part, to the lower protein consumption of sufficiently physically active OW/OB children. On the other hand, PA can reduce uric acid levels even in the absence of weight loss [101], which in turn can reduce TAC levels [22, 72]. In a model that was adjusted for ptn/carbo, the multivariate pattern of HPA explained 38% of the negative variation in TAC in children with excess weight. Therefore, HPA levels may potentially explain the lower TAC levels in sufficiently physically active OW/OB preschoolers. TAC was related with the HPA pattern accumulated during bouts of MVPA. As seen for SOD activity, the association between total time of bouts at MVPA and TAC levels in OW/OB preschoolers likely reflects the accumulated time in bouts of the intensity ranges that were most essential in the multivariate relationship between HPA and TAC.

Interestingly, the most significant intensity range in the association between the multivariate pattern of HPA and TAC was similar to that in the association with SOD activity (8000 to 9999 cpm) and, thus, within the VPA spectrum. In fact, comparing graphs (Fig. 2) they reveal that the multivariate pattern of HPA associated with TAC and SOD activity in OW/OB preschoolers is strikingly similar, but in the opposite direction. Therefore, it is possible that leptin levels in the range of 8000 to 9999 cpm (according to time in HPA) may have reflected changes in TAC levels, but in the opposite direction as observed for SOD activity. In this instance, the lower production of reactive oxygen and nitrogen species, as a result of lower leptin concentrations, may have reduced the need for higher TAC levels in sufficiently physically active OW/OB preschoolers.

To the best of our knowledge, no studies have examined preschoolers when examining the relationship between PA and TAC in obese children. When examining the total index and frequency of PA in organized, non-organized, or competitive sports as well as following an acute bout of aerobic exercise at an intensity corresponding to 70% of their VO2max, studies that looked at obese children between the ages of 8 and 12 found a positive association between TAC and PA [102, 103]. However, the only study that used accelerometry (GT3X) to measure the PA level of children/adolescents (6 to 14 years old) [29], of which 65% were obese individuals, found that individuals classified as highly active (76 ± 17 min of MVPA and 397 ± 54 min of SED) had not only lower TAC, but also lower fat mass compared to moderately active individuals (55 ± 12 min of MVPA and 471 ± 95 min SED). As limitations of the study, the researchers analyzed only the vertical axis of acceleration, did not investigate the multivariate pattern of PA, and did not evaluate children separately according to their BMI. Thus, it is possible that the relationship between PA and TAC in obese children is greatest at the highest intensities of traditional PA. Regarding preschoolers, our findings indicate that the association between HPA and TAC in the OW/OB group occurs primarily at the VPA level. Consequently, it is plausible that the difference in HPA patterns between EU and OW/OB preschoolers explains why we have only observed an association between HPA and TAC in OW/OB preschoolers. Similarly, Leite-Almeida et al. [103] found a association between PA and TAC in obese children only (8–9 years old). Notably, the relationship between PA and TAC was not adjusted for protein consumption in the aforementioned studies [29, 102, 103]. In order to understand the role of high protein consumption in the relationship between PA/HPA and redox status in obese children, further studies are necessary.

The absence of an association between adiposity and triglycerides with HPA levels or traditional intensities of PA may explain the absence of an association between the multivariate pattern of HPA and TBARS in OW/OB preschoolers [104].

### Limitations and future directions

The present study has some limitations. TAC levels from the current study differ from those recorded in previous investigations in children, thus the discrepancies may be explained by the different methodological approaches [65, 66]. In addition, different from other studies that combined prepubertal and preschool-aged children in the sample, our sample consisted solely of preschool-aged children, separated according to BMI and HPA levels, and matched by potential confounding factors. However, our results limit the generalization of the findings because our sample was mostly composed of females, with mothers that had completed high school and were class C economic level.

Another limitation is that the cut-off points for PA intensities for Brazilian preschoolers remain undefined. However, the PLS analysis reduces the dependency on the cut-off points. In addition, the multivariate pattern analysis of HPA allowed us to identify a tighter range of VPA compatible with preschool age. In contrast, accelerometers worn at the waist and PA classification cutoff points do not distinguish whether individuals are sitting or standing [105]. Thus, it is possible that the preschoolers’ sedentary behaviors were included in the LPA analysis [87].

The redox status responses to PA practice depend on the mode and intensity of PA; therefore, it is essential to determine the optimal manner for preschoolers to obtain enough levels of HPA and, consequently, health benefits through improvements in redox balance. In addition, future studies should also verify whether the relationship between HPA and redox status differs in obese preschoolers due to metabolic issues caused by excess weight and/or imposed differences in the pattern of HPA, especially the HPA pattern accumulated in bouts of higher intensities. Moreover, it would be interesting to determine whether a PA threshold-intensity is necessary to trigger oxidative stress responses in preschoolers.

Finally, because this study is cross-sectional in nature, it is impossible to draw cause-and-effect inferences from it. Thus, longitudinal studies are required to verify the effects of HPA on redox status and inflammatory biomarkers in EU and OW/OB preschoolers.

## Conclusion

Our findings indicate that OW/OB preschoolers have higher levels of oxidative stress biomarkers and pro-inflammatory cytokines compared to EU preschoolers. Although EU and OW/OB preschoolers have similar levels of HPA, they have different daily sporadic short-duration physical activities pattern. HPA in the VPA ranges may exert antioxidative and anti-inflammatory effects in OW/OB preschoolers.

### Electronic supplementary material

Below is the link to the electronic supplementary material.

Supplementary Material 1

Supplementary Material 2

Supplementary Material 3

## Data Availability

The datasets used and/or analyzed during the current study are available from the corresponding author on reasonable request.

## Abbreviations

BMI: Body Mass Index
CAT: Catalase
EU: Eutrophic
FMI: Fat Mass Index
FRAP: Ferric Reducing Ability of Plasma
HDL: High Density Lipoprotein
HPA: Habitual Physical Activity
LDL: Low Density Lipoprotein
LMVPA: Light, Moderate to Vigorous Physical Activity
LPA: Light Physical Activity
MDA: Malondialdehyde
MPA: Moderate Physical Activity
MVPA: Moderate to Vigorous Physical Activity
OW: Overweight
OW/OB: Overweight/Obese
PA: Physical Activity
PLS: Partial least squares (regression)
Ptn/carbo: Protein/carbohydrate ratio
SED: Sedentary
SOD: Superoxide Dismutase
SR: Selectivity Ratio
sTNFR1: Soluble Tumor Necrosis Factor Receptor 1
sTNFR2: Soluble Tumor Necrosis Factor Receptor 2
sTNFRs: Soluble Tumor Necrosis Factor Receptors
TAC: Total Antioxidant Capacity
TBARS: Thiobarbituric Acid Reactive Substances
TNF: Tumor Necrosis Factor
VPA: Vigorous Physical Activity
WHO: World Health Organization
