# Supplementary material for: Association between biomarkers of redox status and cytokines with different patterns of habitual physical activity in eutrophic and overweight/obese preschoolers: multivariate analysis of a cross-sectional study
Source: BMC Public Health. 2023 Nov 28;23:2353. doi: 10.1186/s12889-023-17295-y (PMC10683275; doi:10.1186/s12889-023-17295-y)
Supplement: Supplementary file 1 — Supplementary Material 1 [file 12889_2023_17295_MOESM1_ESM.docx]

Supplementary Table S1: Habitual physical activity (HPA) levels of eutrophic and overweight/obese groups.

|  | Sufficiently active | | | | Insufficiently active | | | |
| --- | --- | --- | --- | --- | --- | --- | --- | --- |
|  | EU group | OW/OB group | T | p | EU group | OW/obese group | T | p |
| HPA *(min) m ± sd* |  |  |  |  |  |  |  |  |
| SED | 372 ± 40 | 385 ± 39 | -0.84 | 0.40 | 416 ± 41 | 420 ± 40 | -0.24 | 0.80 |
| LPA | 209 ± 32 | 199 ± 25 | 0.95 | 0.34 | 174 ± 37 | 168 ± 36 | 0.43 | 0.67 |
| MPA | 46 ± 8 | 48 ± 5 | -0.98 | 0.33 | 31 ± 9 | 33 ± 7 | -0.86 | 0.39 |
| VPA | 25 ± 5 | 23 ± 4 | 1.27 | 0.21 | 17 ± 7 | 15 ± 4 | 0.80 | 0.43 |
| MVPA | 71 ± 10 | 71 ± 8 | -0.19 | 0.98 | 47 ± 9 | 48 ± 10 | -0.23 | 0.81 |
| Nº bouts ≥ 2 min | 59 ± 10 | 60 ± 7 | -0.39 | 0.69 | 41 ± 10 | 42 ± 9 | -0.36 | 0.72 |
| Total bouts time ≥ 2 min | 160 ± 28 | 163 ± 18 | -0.33 | 0.74 | 106 ± 26 | 110 ± 25 | -0.31 | 0.75 |
| Average time in bouts ≥ 2 min | 2.6 ± 0.1 | 2.6 ± 0.1 | 0.40 | 0.68 | 2.5 ± 0.1 | 2.5 ± 0.1 | -0.03 | 0.97 |

OW: overweight; m ± (sd): mean ± (standard deviation). Quantitative variables with normal distribution were analyzed by the T test. SED: sedentary behavior. LPA: light physical activity. MPA: moderate physical activity. VPA: vigorous physical activity. MVPA: moderate to vigorous physical activity. Significant difference: p <0.05
