# Supplementary material for: Association between biomarkers of redox status and cytokines with different patterns of habitual physical activity in eutrophic and overweight/obese preschoolers: multivariate analysis of a cross-sectional study
Source: BMC Public Health. 2023 Nov 28;23:2353. doi: 10.1186/s12889-023-17295-y (PMC10683275; doi:10.1186/s12889-023-17295-y)
Supplement: Supplementary file 2 — Supplementary Material 2 [file 12889_2023_17295_MOESM2_ESM.docx]

Supplementary Table S2: Correlation between adiposity, redox status biomarkers, cytokines, and metabolic stressors in insufficiently and sufficiently physically active preschoolers

|  | 1* | 2* | 3 | 4 | 5 | 6 | 7* | 8 | 9 | 10 | 11 |
| --- | --- | --- | --- | --- | --- | --- | --- | --- | --- | --- | --- |
| 1. BMI* |  | **0.88** | **0.82** | **-0.68** | **-0.55** | **0.82** | **0.52** | 0.40 | **0.81** | 0.02 | **0.66** |
| 2. FMI* | **0.92** |  | **0.77** | **-0.63** | **-0.50** | **0.73** | **0.55** | **0.45** | **0.84** | 0.02 | **0.60** |
| 3. TAC* | 0.26 | **0.45** |  | **-0.64** | **-0.47** | **0.52** | **0.44** | **0.44** | **0.59** | -0.01 | 0.40 |
| 4. SOD* | 0.22 | 0.15 | -0.27 |  | **0.89** | -0.28 | **-0.49** | **-0.49** | **-0.48** | 0.41 | -0.32 |
| 5. CAT | 0.00 | 0.07 | 0.09 | 0.36 |  | -0.21 | -0.48 | -0.36 | -0.38 | -0.08 | -0.37 |
| 6. TBARS* | 0.18 | 0.06 | 0.12 | 0.04 | -0.08 |  | 0.34 | 0.36 | **0.66** | **0.40** | **0.73** |
| 7. sTNFR1* | 0.14 | 0.28 | 0.27 | -0.02 | -0.03 | -0.01 |  | **0.72** | 0.31 | -0.16 | 0.20 |
| 8. sTNFR2 | 0.06 | 0.14 | 0.22 | 0.28 | -0.25 | -0.18 | **0.63** |  | 0.31 | -0.12 | 0.20 |
| 9. Leptin* | 0.79 | 0.71 | 0.10 | 0.23 | **0.37** | 0.09 | 0.05 | -0.08 |  | -0.11 | **0.66** |
| 10. Triglycerides* | 0.00 | -0.08 | -0.14 | **0.39** | 0.37 | 0.40 | -0.23 | -0.30 | 0.20 |  | -0.11 |
| 11. Ptn/carbo | 0.21 | 0.04 | -0.19 | 0.17 | 0.31 | 0.06 | -0.25 | -0.07 | 0.15 | 0.23 |  |

Bivariate correlations in insufficiently (top triangle) and sufficiently physically active (bottom triangle) preschoolers. *Variables with non-normal distribution for which Spearman's correlation was performed. BMI: Body Mass Index. FMI: Fat Mass Index. TAC: Total antioxidant capacity. SOD: Superoxide dismutase. CAT: Catalase. TBARS: Substances reactive to thiobarbituric acid. sTNFR1: Soluble Tumor Necrosis Factor Receptors 1. sTNFR2: Soluble Tumor Necrosis Factor Receptors 2. Ptn/carbo: Protein/carbohydrate ratio. Values in bold show correlation coefficients with p value <0.05.
